# Implications of size‐selective fisheries on sexual selection

**DOI:** 10.1111/eva.12988

**Published:** 2020-06-22

**Authors:** Silva Uusi‐Heikkilä

**Affiliations:** ^1^ Department of Biological and Environmental Science University of Jyväskylä Jyväskylä Finland

**Keywords:** fisheries‐induced evolution, inbreeding avoidance, mate choice, mate competition, plastic response, sex‐biased fisheries, size variability

## Abstract

Fisheries often combine high mortality with intensive size selectivity and can, thus, be expected to reduce body size and size variability in exploited populations. In many fish species, body size is a sexually selected trait and plays an important role in mate choice and mate competition. Large individuals are often preferred as mates due to the high fecundity and resources they can provide to developing offspring. Large fish are also successful in competition for mates. Fisheries‐induced reductions in size and size variability can potentially disrupt mating systems and lower average reproductive success by decreasing opportunities for sexual selection. By reducing population sizes, fisheries can also lead to an increased level of inbreeding. Some fish species avoid reproducing with kin, and a high level of relatedness in a population can further disrupt mating systems. Reduced body size and size variability can force fish to change their mate preferences or reduce their choosiness. If mate preference is genetically determined, the adaptive response to fisheries‐induced changes in size and size variability might not occur rapidly. However, much evidence exists for plastic adjustments of mate choice, suggesting that fish might respond flexibly to changes in their social environment. Here, I first discuss how reduced average body size and size variability in exploited populations might affect mate choice and mate competition. I then consider the effects of sex‐biased fisheries on mating systems. Finally, I contemplate the possible effects of inbreeding on mate choice and reproductive success and discuss how mate choice might evolve in exploited populations. Currently, little is known about the mating systems of nonmodel species and about the interplay between size‐selective fisheries and sexual selection. Future studies should focus on how reduced size and size variability and increased inbreeding affect fish mating systems, how persistent these effects are, and how this might in turn affect population demography.

## 1 INTRODUCTION

The high mortality and size selectivity (i.e., removal of large individuals) imposed by many commercial and recreational fisheries may negatively affect population productivity, as small fish (subjected to low fishing mortality) typically have low fecundity and reproductive success (Barneche, Robertson, White, & Marshall, 2018; Shelton, Sinclair, Chouinard, Mohn, & Duplisea, 2006; Uusi‐Heikkilä et al., 2015). An important, but often neglected, factor contributing to reproductive success in exploited fish populations is intersexual (i.e., mate choice) and intrasexual selection (i.e., competition to access the opposite sex, hereafter referred to as “mate competition”). Body size plays an important role in sexual selection in numerous fish species, as females may prefer large males who are superior in male–male competition and have high resource‐holding potential (Huntingford & Turner, 1987; Järvi, 1990; Parker, 1974; van den Berghe & Gross, 1989). Among some species, males exercise mate choice and prefer large females who produce a high number of large eggs (e.g., Passos, Vidal, & D’Anatro, 2019). Sexual selection creates important filters for reproductive success and can consequently increase fitness and enhance population viability (Reynolds & Gross, 1992; Whitlock & Agrawal, 2009).

**FIGURE 1:**
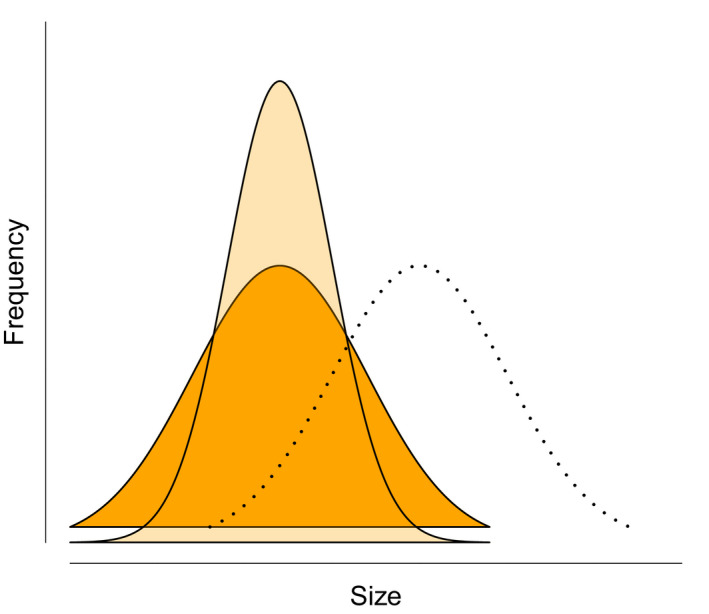
Hypothetical body size distributions of an unfished population (dotted line), an exploited population where the average body size has decreased (orange), and an exploited population where the average body size *and* size variability have decreased (light orange)

Although sexual selection is a powerful selective force (Kingsolver et al., 2001), it cannot necessarily rescue exploited populations (i.e., decelerate fisheries‐induced evolutionary changes) if fisheries have substantially reduced the variability in a sexually selected trait (e.g., body size). Most studies of fisheries‐induced evolution have focused on responses in *average* trait values. The more subtle, yet important, within‐population changes in trait *variability* have received little attention (Figure 1). Olsen et al. (2009) studied coastal Atlantic cod (*Gadus morhua*) populations harvested by both commercial and recreational fisheries. They showed that while the average juvenile body size did not change over time, the size variability decreased substantially (Olsen, Carlson, Gjøsæter, & Stenseth, 2009). Another example comes from an experimental study, where zebrafish (*Danio rerio*) were exposed to size‐selective harvesting (Uusi‐Heikkilä et al., 2015). Fish in a harvest treatment mimicking fisheries selection (i.e., large fish were removed) exhibited lower variability in growth and body size than did fish in an opposite harvest treatment (i.e., small fish were removed) despite no significant treatment‐specific differences in the average size at age (Uusi‐Heikkilä, Lindström, Parre, Arlinghaus, & Kuparinen, 2016).

Reduced trait variability can affect a population's adaptive potential, stability, and resilience (Allendorf, England, Luikart, Ritchie, & Ryman, 2008; Dochtermann & Gienger, 2012). Yet its impact on mating systems in exploited fish populations has been poorly studied (but see Hutchings & Rowe, 2008). Many exploited populations have not recovered despite fishing has been stopped (Hutchings, 2000; Shelton et al., 2006; Worm et al., 2009). These populations likely suffer from reduced average body size, low size variability, and potentially disrupted mating systems, all of which contribute to population decline (Rowe & Hutchings, 2003). However, we know relatively little about the mating systems of many commercially important fish species, which hinders our understanding of the processes enhancing population growth and their potential to recover (Amundsen, 2003; Rowe & Hutchings, 2003).

Although fisheries‐induced changes in phenotypic variability have been less studied, changes in genetic variability have received considerable attention (e.g., Cuveliers, Volckaert, Rijnsdorp, Larmuseau, & Maes, 2011; Pinsky & Palumbi, 2014; Ruzzante, Taggart, Doyle, & Cook, 2001; Therkildsen, Nielsen, Swain, & Pedersen, 2010). Fisheries have been shown to decrease effective population sizes (Hauser, Adcock, Smith, Barnal‐Ramirez, & Carvalho, 2002) and increase inbreeding in exploited populations (Buchholz‐Sørensen & Vella, 2016; Hoarau et al., 2005; O’Leary et al., 2013). An increased level of relatedness in a population can further disrupt mating systems through inbreeding avoidance (i.e., behavioral avoidance of mating with kin; Gerlach & Lysiak, 2006; Mehlis, Bakker, & Frommen, 2008). Inbreeding can lead to inbreeding depression (i.e., reduced fitness of closely related parents’ offspring; Frommen, Luz, Mazzi, & Bakker, 2008; Thrower & Hard, 2009), which can lower population growth and viability (O’Grady et al., 2006; Reed, Lowe, Briscoe, & Frankham, 2003). Yet there have been few studies quantifying the magnitude of inbreeding in exploited fish populations, and our understanding of the strength and demographic consequences of inbreeding avoidance and depression in the wild is still limited (Kardos, Taylor, Ellegren, Luikart, & Allendorf, 2016).

While life‐history responses have been documented in many exploited fish populations (e.g., Rijnsdorp, 1993; Sharpe & Hendry, 2009; Swain, Sinclair, & Hanson, 2007), little is currently known about the evolution of mate choice systems in response to fisheries. If mate preferences are genetically based (Bakker & Pomiankowski, 1995; Chenoweth & Blows, 2006), the choosing sex might not be able to switch its preference rapidly when environmental and social conditions change. However, forming mate preferences is a complex process involving not only genetic factors but also nongenetic ones. Increasing evidence suggests that mate preference can be condition‐dependent and that the social environment is an important factor in forming mate preferences (e.g., Ah‐King & Gowaty, 2016; Lehtonen, Wong, & Lindström, 2010; Meuthen, Baldauf, Bakker, & Thünken, 2019). Accordingly, fish might be able to switch their mate preferences flexibly if the costs of finding a formerly preferred mate become too high. Costly mate choice might lead the choosing sex to switch their preferences or at least to become less discriminative (e.g., Sørdalen et al., 2018). However, we know little of the mechanisms or rate of these processes or how the fisheries‐induced life‐history changes interact with mate choice behavior.

If size‐selective fisheries reduce average body size and size variability in a population (Nusslé et al., 2017), this could have an impact on those mating systems where size is a sexually selected trait. Many fish species base their mate choice on body size, but other traits, such as breeding coloration (Bakker & Mundviler, 1994), age (Brooks & Kemp, 2001), or sound production (Nordeide & Kjellsby, 1999), can also be sexually selected. Here, I focus on body size because fisheries are often selective for size. First, I consider how size‐selective fisheries could modify sexual selection in a way that might affect individual reproductive success and eventually population growth. I then touch upon fishing‐induced changes in sex ratio and their effects on mate choice and mate competition. I move on to discuss what effects increased levels of inbreeding could have on mate choice and individual reproductive success. Finally, I contemplate the potential of plastic adjustments or evolutionary changes in the mating systems of size‐selectively exploited populations.

## 2 THE EFFECT OF SIZE‐SELECTIVE FISHERIES ON SEXUAL SELECTION

### 2.1 Intersexual selection: mate choice

In many fish species, large body size is an adaptation favored by sexual selection. Large individuals may be preferred by the opposite sex because they can provide high‐quality resources, such as nests or territories, genetic advantages to improve offspring quality (“good genes”) or simply have higher fertility (Table 1). Our understanding of fish mate choice is mostly based on model species, and the mating systems of many commercially valuable fish species (e.g., flatfishes) are poorly known (Amundsen, 2003; Auld, Noakes, & Banks, 2019), perhaps excluding the Atlantic cod. In cod, large males possess more sperm, fertilize more eggs, are more dominant, and court females more vigorously (i.e., circle around females) than small males (Hutchings, Bishop, & McGregor‐Shaw, 1999; Trippel & Morgan, 1994). Females likely assess male quality during the circling bouts, and the frequency of male circling behavior has been suggested to play an important role in female mate choice (Hutchings et al., 1999). The removal of large, dominant males by fishing can potentially extend the spawning intervals between egg batches through constant re‐establishment of male dominance ranks (Hutchings et al., 1999). Prolonged spawning can lead to reduced reproductive success because eggs unduly retained in the ovary after ovulation can become unviable or have low egg fertilization probability (Kjesbu, 1989; Kjørsvik & Lønning, 1983; Kjørsvik, Mangor‐Jensen, & Holmefjord, 1990). Furthermore, reduced size variability may translate directly to low variability in courting behavior and this can complicate female assessment of males (Luttbeg, 2002; Mazalov, Perrin, & Dombrovsky, 1996).

**TABLE 1:** Examples of species and potential benefits a large‐sized mate could provide to the choosing sex compared to a small‐sized mate

Benefit for the choosing sex	Choosing sex	Species	Reference
Larger (heavier) nest Better nest concealment Higher paternal quality Higher territory quality	Female	Three‐spine stickleback	Candolin and Salesto (2006) ; Kraak, Bakker, and Mundwiler (1999) ; Rowland (1989)
Higher probability to obtain a nest	Female	Sand goby	Magnhagen and Kvarnemo (1989)
Higher offspring quality	Female	Guppy	Reynolds and Gross (1992)
Higher sperm number	Female	Guppy Mosquitofish Cod	Pitcher and Evans (2004) ; O’Dea, Jennions, and Head (2014) ; Trippel and Morgan (1994)
Less harassment from other males Better distraction of predators	Female	*Brachyrhapsis rhabdophora*	Basolo (2004)
More intensive parental care	Female	Smallmouth bass	Sutter et al. (2012) ; Wiegmann and Baylis (1995)
Lower rate of egg cannibalism	Female	Brook charr Sculpin Red‐lipped blenny	Blanchfield and Ridgway (1999) ; Downhower and Brown (1980) ; Cóte and Hunte (1989)
Higher probability to provide a shelter for molting and mating	Female	American lobster	Karnofsky and Price (1989)
Higher fecundity Heavier eggs	Male	Three‐spine stickleback Pacific blue‐eye Pipefish	Candolin and Salesto (2006); Kraak and Bakker (1998) ; Wong and Jennions (2003) ; Rosenqvist (1990)
Better access to nesting habitats Better quality nests (deeper redds) More intensive nest defense	Male	Pacific salmon	Fleming and Gross (1994) ; Steen and Quinn (1999) ; Van den Berghe and Gross (1984)

In zebrafish, females prefer large males for potentially two reasons: Large males are better able to acquire and defend territories than small males are (Spence & Smith, 2005), and because of the genetic contribution to the developing offspring (Uusi‐Heikkilä, Kuparinen, Wolter, Meinelt, & Arlinghaus, 2012). Independently of female body size, the eggs fertilized by large zebrafish males had higher hatching probability and larvae hatching from those eggs were larger than were the ones fertilized by small males (Uusi‐Heikkilä, Kuparinen, et al., 2012). When same‐sized zebrafish females were coupled with either a large or a small male, females spawned more frequently with large males (Uusi‐Heikkilä, Böckenhoff, Wolter, & Arlinghaus, 2012). Females also differentially allocated their reproductive resources toward larger males by producing larger egg batches for them (Uusi‐Heikkilä, Böckenhoff, et al., 2012). This pattern was independent of the past social environment females had experienced.

In addition to zebrafish, differential allocation has also been demonstrated in swordtail fish (*Xiphophorus multilineatus*; Rios‐Cardenas, Brewer, & Morris, 2013), Banggai cardinal fish (*Pterapogon kauderni*; Kolm, 2001; Kolm & Olsson, 2003), and in sand goby (*Pomatoschistus minutus*; Lehtonen & Lindström, 2007). When chinook salmon (*Oncorhynchus tshawytscha*) females were paired with a small male, they delayed spawning allowing larger males the opportunity to displace the small courting male (Berejikian, Tezak, & LaRae, 2000). Similar behavior was reported in chum salmon (*O. keta*; Schroder, 1981). Manipulation of the spawning duration can be a common female response to variation in male size and could represent a form of differential allocation (e.g., Kolm & Olsson, 2003; Makiguchi et al., 2016). Altogether, these examples illustrate that at least in some species male body size can indirectly increase reproductive success. Differential allocation can be facilitated through an extended pair bond or by the ability of females to adjust their egg investment after the onset of egg maturation (Kolm & Olsson, 2003). In teleost fishes, the period when proteins are packed into the oocytes can be rather short (Koya, Itazu, & Inoue, 1998). Therefore, females are potentially able to control egg investment after maturation in response to the attractiveness of their current mate.

### 2.2 Intrasexual selection: mate competition

In addition to mate choice, sexual selection is mediated by mate competition, where body size also plays an important role. In competitive male–male interactions, large males frequently have an advantage over small suitors (Andersson, 1994; but see Qvarnström & Forsgren, 1998). In cod, large males are more dominant and obtain access to females more frequently than small males do (Hutchings et al., 1999). Male cod and haddock (*Melanogrammus aeglefinus*) are known to attract females by producing a “drumming” sound (Hawkins, 1986; Hutchings et al., 1999), which is also a feature in male–male aggression (Hawkins, 1986). The volume of drumming muscles increases with body size (Hutchings et al., 1999) and males with larger drumming muscles have higher fertilization potential (Engen & Folstad, 1999). Anadromous Atlantic salmon (*Salmo salar*) males typically form dominance hierarchies in which one or a few individuals control access to females (Fleming, 1996). They invest heavily in resources for searching and fighting for mates and courting them. The largest and more dominant males are predicted to have the highest reproductive success (Fleming, 1996; Järvi, 1990).

When male size and size variability are reduced, females will encounter the large, preferred mating partners less frequently. This can lead to increased costs of mate choice, as females must allocate more time and energy to find a suitable mate (Crowley et al., 1991; Real, 1990) and might become exposed to predation (Forsgren, 1992; Godin & Briggs, 1996). Consequently, females may become less choosy. For example, female two‐spotted goby (*Gobiusculus flavescens*) have been shown to prefer large males early in the breeding season but reduce their choosiness as the breeding season progresses (Borg, Forsgren, & Amundsen, 2006). This could be due to the high costs associated with mate searching as male availability decreases toward the end of the breeding season. The two‐spotted goby has adapted to natural temporal variation in sex ratio and, therefore, it is reasonable to expect its mate choice to be variable (Borg et al., 2006). Fishing can change the social environment rapidly and unpredictably. This change results in novel conditions that the species has not encountered during its evolutionary past (see also Sih, Ferrari, & Harris, 2011; Tuomainen & Candolin, 2011), thereby compromising the adaptive value of body size in attracting and selecting mates. Consequently, individuals might not be able to alter their choosiness as flexibly and rapidly as can those species adapted to seasonal variation in mate availability.

If males are both small and in poor condition, females might not allocate time to inspect these mates closely (Candolin, 2019). In Atlantic cod, females typically initiate and terminate the male courting behavior (Hutchings et al., 1999). Large males have been shown to circle around females more frequently than small males do, potentially allowing better evaluation by females (Wright & Rowe, 2018). Although not shown by Hutchings et al. (1999), females potentially might terminate the courting behavior of small males sooner than that of large males because they choose not to allocate more time inspecting small males. In Eastern Baltic Sea cod, male body size and condition have decreased substantially during the last few decades of intensive fishing and unfavorable environmental conditions (Casini et al., 2016; Eero et al., 2015). It is not known whether the decline in male size and condition has affected female mate choice or male courting behavior in this particular stock. Despite heavy reductions in fishing pressure, the Eastern Baltic Sea cod stock has not recovered (ICES, 2019). Certain exploited populations have failed to recover because of the phenomenon known as the Allee effect: Decrease in population size appears to have been associated with a decline in per capita population growth rate (Hutchings, 2014; Hutchings & Kuparinen, 2014). One underlying mechanism of the Allee effect could be disrupted mating systems (Dennis, 1989) due to, for example, reduced body size and condition of the courting sex. Allee effect in small populations can limit their growth, slow their recovery, and increase the uncertainty of their recovery time (Kuparinen, Keith, & Hutchings, 2014).

In an unexploited population characterized by a breadth of phenotypic variability, there are typically asymmetries between competing individuals (contestants). These important asymmetries include, for example, fighting ability and body size. Body size is one of the most important factors in determining contest outcome in many fish species (Huntingford & Turner, 1987). In cichlids, the eventual winners and losers of fights can be predicted by the size or weight of the contestants (Huntingford, Taylor, Sneddon, & Neat, 2001). Differences of as little as 2% in weight can be enough to predict the winners (Barlow, Rogers, & Fraley, 1986; but see Neat, Huntingford, & Beveridge, 1998). Opponents and bystanders can assess each other's size and strength and use that information in decision‐making (Peake & McGregor, 2004). For example, tail beating can provide information of the contestant's body size to the opponent as well as to bystanders (Hurd, 1997).

If size‐selective fisheries reduce size variability in a population, important asymmetries that fish use in decision‐making in competitive situations cease to exist. Consequently, contests can become prolonged and intense (Barrette & Vandal, 1990; Jonart, Hill, & Badyaev, 2007; Parker & Rubenstein, 1981) because males cannot distinguish the competitive quality of the conspecific and potentially adopt an alternative behavioral strategy (“flight or fight”). This can further disrupt female mate choice, as females cannot easily detect a male's perceived quality (Luttbeg, 2002; Mazalov et al., 1996). The disruption of mate competition, and consequently, mate choice might have various consequences for reproduction. Females might skip spawning entirely (Trippel & Harvey, 1990) or spawning intervals between egg batches can be prolonged (Hutchings, Bishop, & McGregor‐Shaw, 1999), potentially causing low fertilization success due to over‐ripening of gametes (Kjørsvik et al., 1990). Alternatively, it can lead to differential allocation, where females produce lower quality embryos to nonpreferred males (Kolm, 2001; Kolm & Olsson, 2003). Ultimately, this can affect population growth rate negatively and hinder population recovery after fishing has ceased.

### 2.3 Fisheries‐induced changes in sex ratio

Fisheries can bias population sex ratio if one sex is harvested more intensively (Fenberg & Roy, 2008; Ginsberg & Milner‐Gulland, 1994; McCleave & Jellyman, 2004). The sockeye salmon (*O. nerka*) fisheries in Bristol Bay, Alaska, have disproportionally exploited more males than females (Kendall, Hard, & Quinn, 2009; Kendall & Quinn, 2009, 2012). Males are more vulnerable to gillnetting because of their larger size, greater body depth, and bigger jaws and teeth (Kendall & Quinn, 2012). The higher proportion of females compared to males altered male–male interaction and behavior: Sockeye salmon males became less competitive and more mobile in the presence of excess females (Mathisen, 1962). A female‐biased sex ratio is predicted to relax selection on males and provide opportunities to those males that might otherwise be dominated by large males in competition and not favored by females (Foote, 1989; Quinn, Hendry, & Buck, 2001; Rowe & Hutchings, 2003). In sand gobies, a female‐biased sex ratio allowed even small males to build nests and spawn (Kvarnemo, Forsgren, & Magnhagen, 1995). Thus, a skewed sex ratio can alter reproductive behavior, including nest building, courtship, aggression, and individual reproductive success.

Fisheries that selectively remove larger males from a population favor small males. Those are able to escape fisheries and experience reduced mate competition with larger males on the breeding ground. Size at maturation is heritable in salmonids (e.g., Barson et al., 2015), so as the reproductive success of smaller males increases, the size of individuals in future generations may decrease. In female‐biased populations with a high proportion of young males, females might hesitate to breed with nonpreferred males (Berejikian et al., 2000; de Gaudemar, Bonzom, & Beall, 2000). This can lead to differential reproductive allocation or extend the spawning period (as described in previous chapters). Despite the skewed sex ratio, Bristol Bay sockeye have thrived and no clear changes in population productivity have been detected (Hilborn, Quinn, Schindler, & Rogers, 2003). Indeed, it has been predicted that if females in general are less heavily exploited, the impact on population growth and sustainability might be small (Ginsberg & Milner‐Gulland, 1994; Hays, Mazaris, Schofield, & Laloë, 2017). However, in a broadcast spawning species, such as Atlantic cod, a female‐biased sex ratio can lead to a low egg fertilization rate and high variance in fertilization success (Rowe, Hutchings, Bekkevold, & Rakitin, 2004). A large number of males circling the focal female were suggested to ensure high sperm concentration and fertilization success (see also Shapiro, Marconato, & Yoshikawa, 1994). Thus, it is difficult to generalize or predict the outcome of changed sex ratio without a proper understanding of the mating system in question.

Unlike in Bristol Bay sockeye, some fisheries target large females and create male‐biased sex ratio. For example, spiny dogfish (*Squalus acanthias*) landings on the east coast of the United States consist mostly of females (Haugen, Curtis, Fernandes, Sosebee, & Rago, 2017; NEFSC, 2006). Similarly, the southern flounder (*Paralichthys lethostigma*) fishery along the south Atlantic coast of the United States is heavily dependent on large females (Fitzhugh, Crowder, & Monaghan, 1996). These particular studies did not describe the effect of a male‐biased sex ratio on female mate choice but demonstrated how a reduction in the proportion of females might translate into a reduction in recruitment to the fishery (Honeycutt et al., 2019). A male‐biased environment may not change female interactions toward males but it might change male–male interactions (Kvarnemo et al., 1995). In a male‐biased sand goby population, nest‐building males were larger compared to those not building a nest, likely because intense male–male competition made small males refrain from building nests (Kvarnemo et al., 1995). Although most of the females likely had the opportunity to reproduce because the male‐biased sex ratio supported the reproductive success of females, the generally low number of females could have reflected negatively on population growth (as suggested in the southern flounder example, see Honeycutt et al., 2019). Skewed sex ratios can affect breeding dynamics and sexual selection, with the potential for both ecological and evolutionary consequences. Therefore, understanding the effects of size‐selective fishing on sex ratios may help to explain changes in the structure and sustainability of exploited fish populations. It is important to assess the size dimorphism in exploited populations and consider whether it could interact with selective fishing and result in skewed sex ratios.

### 2.4 The effect of inbreeding on mating systems

Fisheries‐induced genetic bottlenecks were initially thought to be rare because many marine fish species have large population sizes and are connected by larval and adult‐mediated dispersal (Hauser, Adcock, Smith, Barnal‐Ramirez, & Carvalho, 2002; Kenchington & Heino, 2002). Indeed, some studies have shown no effect of harvesting on genetic variability as population sizes declined (Cuveliers et al., 2011; Jakobsdóttir et al., 2011; Poulsen, Nielsen, Schierup, Loeschcke, & Grønkjær, 2006; Ruzzante et al., 2001; Therkildsen et al., 2010). However, others have demonstrated fisheries‐induced decay in genetic diversity or increased inbreeding (Hoarau et al., 2005; Hutchinson, van Oosterhout, Rogers, & Carvalho, 2003; Pinsky & Palumbi, 2014; Smith, Francis, & McVeagh, 1991). For example, North Sea plaice (*Pleuronectes platessa*), winter flounder (*Pseudopleuronectes americanus*) in New York estuaries, and endangered dusky grouper (*Epinephelus marginatus*) in the central Mediterranean have been heavily exploited and consequently experienced moderate to severe inbreeding (Buchholz‐Sørensen & Vella, 2016; Hoarau et al., 2005; O’Leary et al., 2013). Inbreeding can affect population productivity and viability through inbreeding depression (i.e., reduced survival and fertility of offspring of related parents), which animals can avoid through behavioral adaptations, such as inbreeding avoidance.

To avoid breeding with close relatives, individuals first need to recognize kin and then reject them as mates. Three‐spine stickleback (*Gasterosteus aculeatus*) females can recognize their relatives using olfactory cues and adjust their behavior accordingly (Mehlis et al., 2008). Time spent near a male is a good indicator of a mating preference in this species (Milinksi et al., 2005), and it was shown that females spent a significant proportion of their time near a nonrelated male compared to a related male (Mehlis et al., 2008). In this study, inbreeding itself did not affect female preference behavior as both inbred and outbred females spent more time near nonrelated males. However, some studies have shown that inbred individuals can lose their ability to recognize kin (Frommen, Mehlis, Brendler, & Bakker, 2007; Reid, Arcese, & Keller, 2006).

In zebrafish, kin recognition is based on phenotype matching and the preference for kin changes with maturity (Gerlach & Lysiak, 2006; Tang‐Martinez, 2001). Juveniles prefer kin because shoaling with relatives early in life may increase inclusive fitness (e.g., Krause & Ruxton, 2002) but mature females prefer unrelated males indicating inbreeding avoidance (Gerlach & Lysiak, 2006). Females can also avoid inbreeding through postcopulatory mechanisms. Least killifish (*Heterandria formosa*) females have been shown to express postcopulatory inbreeding avoidance and reduce the amount of sibling sperm in their reproductive system compared to nonsibling males (Ala‐Honkola, Tuominen, & Lindström, 2010; but see Gasparini, Congiu, & Pilastro, 2015). In this species, females invest substantially more in each offspring after fertilization than males do and therefore females were expected to avoid inbreeding more, although the authors could not rule out the possibility that males invested less sperm in sibling females (Ala‐Honkola et al., 2010). Intensive size‐selective fisheries not only reduce the amount of preferred mates by removing large individuals from the population but also may concurrently increase the proportion of nonpreferred mates (i.e., close relatives; Buchholz‐Sørensen & Vella, 2016; Hoarau et al., 2005; O’Leary et al., 2013). This can lead, at least among some species, to individuals rejecting mating partners who are related to circumvent the potential costs of inbreeding.

In an exploited population where the rate of relatedness is high, inbreeding avoidance can inflict costs in terms of lost breeding opportunities (Shikano, Chiyokubo, & Taniguchi, 2001). In that case, it might be a better strategy to breed with a relative than not breed at all. This could further accelerate inbreeding and affect population productivity due to inbreeding depression. In guppies, inbreeding has been shown to lead to a significant decline in male sperm number (Zajitschek & Brooks, 2010) reducing fertility. In steelhead (*O. mykiss*), inbreeding caused significant decreases in body length, weight, juvenile survival, and delayed spawning (Naish, Seamons, Dauer, Hauser, & Quinn, 2013; Thrower & Hard, 2009). Similarly, in chinook salmon, inbreeding delayed spawn timing (Waters et al., 2020), which has been suggested to reduce reproductive success in this species (Anderson, Faulds, Atlas, & Quinn, 2013; Sard et al., 2015). Although inbreeding can have negative fitness consequences, it does not always lead to predictable outcomes (Boakes, Wang, & Amos, 2007; Waters et al., 2020) and the relationship between the degree of inbreeding and fitness is often unknown (Lacy, Alaks, & Walsh, 1996; O’Grady et al., 2006). Challenges in estimating the effects of inbreeding lie in obtaining precise estimates of individual inbreeding coefficients (Waters et al., 2020) and fully understanding the phenotypic and demographic effects of inbreeding (Kardos et al., 2016).

It has been suggested that species with communal mating, such as Atlantic cod and haddock, might not be able to discriminate and avoid related individuals (Trippel et al., 2009). Spawning experiments with haddock suggest that mating among siblings may be common in this species, at least in captivity, challenging the idea that kinship plays a role in mate selection (Trippel et al., 2009). Haddock, unlike salmon, stickleback, or zebrafish, disperse their embryos in ocean currents, providing little scope for kin recognition development. However, familiarity is not always required for kin recognition because some fish species are able to recognize unfamiliar kin through self‐reflectance (Hauber & Sherman, 2001; Thünken, Bakker, & Baldauf, 2014). In small populations, the probability of encountering kin and inbreeding may increase even in species with high embryo dispersal. Spawning with kin and inbreeding depression (Gjerde, Gunnes, & Gjedrem, 1983; Kincaid, 1976) have been suggested to be potential mechanisms of the Allee effect.

### 2.5 Fisheries‐induced evolution of mate choice systems

Life‐history and behavioral changes have been demonstrated among several size‐selectively harvested fish populations (e.g., Olsen et al., 2004; Swain et al., 2007; Uusi‐Heikkilä et al., 2015), but it is unknown how adaptive their mate choice behavior is. This depends on the heritability of mate preference or mate choice and the rate at which size variability is being eroded by fishing. It has been suggested that there is an appreciable level of heritable variation in female mate choice behavior within populations (e.g., Bakker & Pomiankowski, 1995). Female guppies, for example, are known to prefer ornamented males and a component of female choosiness (female responsiveness) was shown to be heritable (Brooks & Endler, 2001). Male guppies prefer large females, and individual male mating effort and mating preference were significantly repeatable (although not unanimous; Godin & Auld, 2013). In a recent study, Svensson and colleagues (2017) demonstrated that female preference for male breeding coloration in a sympatric cichlid species pair is influenced by relatively few major genes or genomic regions. They also showed that the female preference did not change after a successful mating with a nonpreferred species (Svensson et al., 2017). A prerequisite for mating preference to evolve in an exploited fish population is within‐population variability in preference (Jennions & Petrie, 1997; Widemo & Sæther, 1999). Intensive, size‐selective fisheries likely reduce phenotypic and genetic variability but it can only be speculated to what extent it might reduce variability related to traits relevant to the mate choice process (e.g., preference, responsiveness, discrimination).

If fish were not able to adjust their mate choice, their original mate preference would become maladaptive (as originally preferred mates are rare). However, it has been shown that mate choice is plastic and context dependent (Cotton, Small, & Pomiankowski, 2006; Hunt, Brooks, & Jennions, 2005; Qvarnström, 2001). Females have been shown to adjust their sampling effort based on conspecific factors. For example, the lower the male density, the less discriminating the females are (Borg et al., 2006; Kokko & Rankin, 2006; Lindström & Lehtonen, 2013). Female European lobsters typically prefer to mate with large males with relatively large claws but when large males were harvested, females mated with smaller males (Sørdalen et al., 2018; see also Gosselin, Sainte‐Marie, & Bernatchez, 2003).

Heterospecific factors can as well be important in shaping mate choice behavior. Sand goby females prefer large and brightly colored males (Forsgren, 1992). However, when predation risk by cod increased, females became less choosy and spent as much time close to small and dull males as they did to large and colorful ones (Forsgren, 1992). Changes in abiotic conditions can also affect mating systems. For example, water turbidity and eutrophication can interfere with mate choice and relax sexual selection. In Lake Victoria, female cichlids of a sympatric species pair preferred conspecific males to heterospecific ones in clear water but mated indiscriminately when water turbidity increased and the color differences of males were masked (Seehausen, van Alphen, & Witte, 1997). Finally, there might be intrinsic factors, such as age, determining mating decisions. Size‐selective fisheries truncate population age structure (e.g., Barnett, Branch, Ranasinghe, & Essington, 2017; Berkeley, Hixon, Larson, & Love, 2004; Ottersen, 2008); therefore, exploited populations consist mostly of young fish. It has been shown that young females can be less flexible in their mate preferences than older females are (Tinghitella, Weigel, Head, & Boughman, 2013). In this study, lower flexibility among young individuals was likely caused by less experience with the social mating environment compared to old females. When the social environment is changing rapidly and unpredictably, the lack of flexibility might become disadvantageous. Life‐history theory predicts that young individuals can afford to be choosy, but as individuals approach the end of their reproductive lives, they should become less choosy because fewer opportunities for mating remain (Real, 1990; see also Kodrick‐Brown & Nicoletto, 2001). Fisheries might not allow for a long reproductive life; thus, it is possible that age is no longer an important determinant of mating decisions, as all individuals need to achieve some reproductive success before being captured.

When size‐selective fisheries reduce body size variability rapidly and unexpectedly, an individual's ability to discriminate among mates can be suddenly hampered (e.g., Francis & Barber, 2013). Size may become an unreliable indicator of male quality or difficult to evaluate; thus, females may benefit from shifting their attention to other cues (e.g., behavior, courtship display) that are easier to evaluate (de Jong, Amorim, Fonseca, & Heubel, 2018; Sbragaglia et al., 2019). However, other potential traits under sexual selection might correlate with body size, and variability in these traits may be reduced together with size variability. For example, several behavioral traits indicating good mate quality and used by females as cues for mate choice, such as aggression or nest guarding behavior, correlate with body size (Table 1). When variability in these traits is reduced, they may become equally difficult to evaluate. Shifting cues can increase the costs of mate evaluation in terms of time, energy, and predation risk, or result in more maladaptive choices (Candolin, 2019). An alternative to shifting cues is to take in new cues. However, perceptual restrictions may prevent the adoption of new cues (e.g., Seehausen et al., 2008) and their evolution may be too slow to rescue mating systems in a rapidly changing environment (Horth, 2007).

## 3 CONCLUSIONS

Reduced size variability can be expected to disrupt mate choice and mate competition in systems where body size is a sexually selected trait. Given that size‐selective harvesting reduces size variability in exploited populations (Hutchings & Baum, 2005; Nusslé et al., 2017), fisheries‐induced changes in phenotypic variability should be routinely highlighted together with changes in mean trait values. When the frequency of large females and males is reduced by fishing, finding an optimal (large) mating partner may become costly. Breeding with a suboptimal (small and/or related) mate can lead to reduced offspring fitness or the choosing sex to allocate lower quality reproductive resources to their mate. A strong mate preference can decrease the probability of finding a suitable mating partner, thus decreasing reproductive success and even leading to an Allee effect (Kokko & Rankin, 2006; Møller & Legendre, 2001). Therefore, it would be important to increase our understanding of the potentially complex mating systems of commercially important fish species and aim to integrate that knowledge in fisheries management.

Small exploited populations may foster inbreeding via mating among relatives (Keller & Waller, 2002). As research methods improve and interest intensifies, studies focusing on inbreeding, its effects on mate choice behavior (inbreeding avoidance) and reproductive success (inbreeding depression) should become more commonplace in commercially important populations. Recent genomic advances enable measuring of inbreeding with great precision, and this could fundamentally alter our understanding of inbreeding in wild populations. Inbreeding can have long‐term effects on population productivity, viability, and ability to evolve in response to stochastic environmental events (Charlesworth & Charlesworth, 1987; Hedrick & Kalinowski, 2000; Keller & Waller, 2002). Therefore, fisheries‐induced changes in population size, genetic variability, and levels of inbreeding should be associated with demographic data, including population growth.

Fisheries present a relatively novel contemporary selection force that changes the environment rapidly by removing individuals that would otherwise likely be favored by natural and sexual selection. However, studies combining the often opposing effects of fisheries and sexual selection are rare (but see Hutchings & Rowe, 2008; Sbragaglia et al., 2019; Sørdalen et al., 2018). Reduced size variability can complicate mate discrimination and decision‐making in competitive interactions weakening the potential for sexual selection (Hutchings & Rowe, 2008; Urbach & Cotton, 2008). Therefore, sexual selection cannot always be expected to buffer the effects of fisheries selection or rescue exploited populations. Disrupted mating systems due to the loss of variability can ultimately lower population productivity and resilience (Lipcius & Stockhausen, 2002; Rowe & Hutchings, 2003) and change population dynamics by affecting species interactions and community structure (Candolin, 2019).

## Data Availability

Data sharing is not applicable to this article as no new data were created or analyzed in this study.Box 1. Thank you Louis for believing in me. After finishing my MSc thesis in a smallish Finnish University, I was confident that I immediately get a permanent position in a nonprofit governmental institute where I could start working as a fish/conservation biologist. That time my, like many other fish biologists’, dream was to work in the field with iconic species, such as Atlantic salmon or brown trout. However, I ended up working with rainbow trout. To be more specific, with dead rainbow trout. After one year in a fish factory, I applied a traineeship in the field of Arctic Research. I ended up in Louis’ laboratory in Québec City. My job was to help PhD students and postdocs in the laboratory and I quickly realized that I had found my calling. During the weekends, I studied population genetics and tried to understand the fundamentals behind extracting DNA from salmon adipose fins, picking out whitefish eggs, and taking care of stickleback families. The year I spent in Louis’ laboratory changed my world. No longer desired I to carry out other peoples’ plans, I wanted to create ideas myself. I fell in love with science and developed a burning desire to start a career. All thanks to Louis! When I finally got a PhD student position back in Europe, I had to say goodbye to Louis, the wonderful working environment and to the brilliant people. I am ever so grateful to Louis that he open‐mindedly gave me an opportunity to work in his laboratory and kicked off my career. I found something that I was truly passionate about—and I still am. Thank you Louis and Happy Birthday!
